# Impact of High-Altitude Hypoxia on Early Osseointegration With Bioactive Titanium

**DOI:** 10.3389/fphys.2021.689807

**Published:** 2021-11-18

**Authors:** Yarong Wang, Zekun Gan, Haibin Lu, Ziyi Liu, Peng Shang, Jian Zhang, Wuwei Yin, Hongxing Chu, Renlei Yuan, Yingxin Ye, Pei Chen, Mingdeng Rong

**Affiliations:** ^1^Department of Periodontology and Implantology, Stomatological Hospital, Southern Medical University, Guangzhou, China; ^2^Department of Implantology, Stomatological Hospital, Southern Medical University, Guangzhou, China; ^3^College of Animal Science, Tibet Agriculture and Animal Husbandry University, Linzhi, China; ^4^Linzhi People’s Hospital, Linzhi, China

**Keywords:** osseointegration, bioactive titanium, hypoxia, normoxia, osteoblast

## Abstract

Nowadays, the bone osseointegration in different environments is comparable, but the mechanism is unclear. This study aimed to investigate the osseointegration of different bioactive titanium surfaces under normoxic or high-altitude hypoxic environments. Titanium implants were subjected to one of two surface treatments: (1) sanding, blasting, and acid etching to obtain a rough surface, or (2) extensive polishing to obtain a smooth surface. Changes in the morphology, proliferation, and protein expression of osteoblasts on the rough and smooth surfaces were examined, and bone formation was studied through western blotting and animal-based experiments. Our findings found that a hypoxic environment and rough titanium implant surface promoted the osteogenic differentiation of osteoblasts and activated the JAK1/STAT1/HIF-1α pathway *in vitro*. The animal study revealed that following implant insertion in tibia of rabbit, bone repair at high altitudes was slower than that at low altitudes (i.e., in plains) after 2weeks; however, bone formation did not differ significantly after 4weeks. The results of our study showed that: (1) The altitude hypoxia environment would affect the early osseointegration of titanium implants while titanium implants with rough surfaces can mitigate the effects of this hypoxic environment on osseointegration, (2) the mechanism may be related to the activation of JAK1/STAT1/HIF-1α pathway, and (3) our results suggest the osteogenesis of titanium implants, such as oral implants, is closely related to the oxygen environment. Clinical doctors, especially dentists, should pay attention to the influence of hypoxia on early osseointegration in patients with high altitude. For example, it is better to choose an implant system with rough implant surface in the oral cavity of patients with tooth loss at high altitude.

## Introduction

The theory of “osseointegration” has existed for more than 50 years (Lee and Bance, 2019). Several basic and clinical studies have confirmed that formation of the osseointegration interface of dental implants is the basis for successful implant restoration (Chen et al., 2019; Lee and Bance, 2019). Although the current success rate of implant restoration in plains and flatlands is relatively high, approximately 1–2% of patients experience implant failure due to the lack of binding at the initial stage of implant insertion (Guglielmotti et al., 2019). Meanwhile, the hypoxic atmosphere characteristic of high-altitude environments in plateau regions is associated with a higher risk of osteoporosis and a longer healing time of bone fractures in the elderly population compared to those from regions of low altitudes, such as plains (Bernardi et al., 2020; Feng et al., 2020). This suggests that oxygen concentration is an important factor for bone defect repair. However, to date, the success rate of oral planting and relevant clinical guidelines in plateaus remains unreported. Thus, the only available clinical guidelines related to planting are specific to plains regions with constant oxygen (Guglielmotti et al., 2019). In this study, the osteogenesis of implants in different environment was evaluated in Xizang, China (highland zone, altitude near 3,000m, severe cold; Liu et al., 2021) and Guangzhou, China (plain zone, altitude near 1,000m, Warm and rainy; Liu et al., 2020a).

Bone defect repair is a complex and multifactorial interactive process (Mavrogenis et al., 2009; Dashnyam et al., 2019). Bone trauma and bone defects lead to local vascular rupture followed by bleeding, which leads to tissue ischemia, hypoxia, and acidosis (Ma et al., 2020; Wu et al., 2020a). This pathological process is not conducive to the growth and repair of bone tissue; thus, the application of tissue engineering to repair and restore bone defects has become a popular and important research topic (Wu et al., 2020b). Current research is focused on establishing a suitable microenvironment for the growth and proliferation of osteoblasts in bone defects and promoting the growth and differentiation of new bone to ultimately form a normal structure with normal function (Bernardi et al., 2020; Feng et al., 2020; Wen and Lv, 2020).

Autogenous bone grafts are typically the first choice of material for use on large bone defects; however, their application is restricted due to limited sources, the risk of infection, and adverse immune responses (Gonzaga et al., 2019; Brunello et al., 2020). Titanium and titanium alloys are widely used in the medical field, particularly in orthopedics due to their stable chemical properties, accessibility, and excellent resistance to corrosion (Lee et al., 2017; Talley et al., 2018). Meanwhile, physical, chemical, and biological surface modification methods can improve the roughness of titanium mesh and promote the adhesion and proliferation of osteoblasts (Lee et al., 2017; Talley et al., 2018). However, research on the effects of modified materials under high-altitude hypoxia is still in the exploratory stages.

In the present study, the microenvironment of bone defects was simulated by establishing a hypoxia model and bone defect model of the rabbit tibial plateau. We aimed to observe and compare osteoblasts cultured on titanium disks with different surfaces under normoxic or hypoxic conditions. The effect of these conditions on the morphology, proliferation, and repair of bone defects has the potential to provide a theoretical basis for the clinical application of titanium plate modification in the treatment of bone defects under normoxic or hypoxic conditions.

## Materials and Methods

### Titanium Plate Preparation

Pure industrial TA2 titanium disks (TA2, Baoji, Shanxi Province, China) were customized (10mm diameter and 2mm thickness) and divided into two groups based on their altered surface (smooth and rough) for evaluation. To obtain a smooth surface, the disks were gradually polished using 600#, 800#, 1,200#, and 1,500# metallographic sandpaper and washed in an ultrasonic bath. To obtain a rough surface, the disks were sandblasted using alumina particles (90–250μm in diameter, at 4.5kPa and 90°C for 30s) and treated with a mixture of 18% hydrochloric acid and 49% sulfuric acid at 60°C for 40min to complete the acid etching process.

### Cell Culture and Establishment of the Hypoxia Model

MG-63 cells (Sigma, United States) were cultured in minimum Eagle’s medium (MEM, Gibco, NY, United States) supplemented with 10% high-quality fetal bovine serum (FBS, Gibco, United States) and 1% penicillin-streptomycin antibiotic solution (Beyotime, Beijing, China). The culture medium was replaced every 2–3days. Once the cells reached 80% confluence, they were passaged at a ratio of 1:3 and incubated with 5% CO2 at 37°C in a saturated humidity incubator (Sanyo, Toshima-ku, Japan). The MG-63 cell suspension was collected and its concentration adjusted to 1×10^5^ cells/ml. A titanium plate was placed in a 48-well plate (Corning, NY, United States), and 100μl of cell suspension was added to the surface of each disk. The plate was then incubated for 3h, and cell adherence to titanium was confirmed by scanning electron microscopy (SEM). Following cell adherence, 400μl of culture medium was added to each well. The 48-well plate was then transferred into a portable hypoxia cell culture device (Billups-Rothenberg, NY, United States) and continuously supplied with mixed gas (90% N_2_, 5% CO_2_, and 5% O_2_). The hypoxia device was placed in an incubator at 37°C to maintain saturated humidity levels. In osteogenic condition, the osteogenic induction medium was added (MEM with 10% FBS, 10mmol/L, β-glycerophosphate, 10nmol/L dexamethasone, and 50μg/mL ascorbic acid).

### Cell Proliferation Assay

MG-63 cell proliferation was determined using the Cell Counting Kit-8 (CCK-8, Sigma, MO, United States) and EdU assay (Sigma). For the CCK-8 assay, the MG-63 cell suspension was adjusted to 1×10^5^ cells/ml and 100μl was seeded on a titanium plate in a 48-well plate. Following adherence, the cells were cultured under normoxia and hypoxia conditions for 24 and 48h. When the hypoxia treatment reached the corresponding time point, the CCK-8 assay was carried out according to the manufacturer’s protocol. Briefly, the CCK-8 solution was added to each well and incubated for 3h. Absorbance was measured at 450nm using a microplate reader.

An EdU assay was performed by treating 5×10^3^ MG-63 cells with 50mM EdU at 37°C for 6h. The cells were stilled attached to the titanium in this assay. After fixation in 4% paraformaldehyde, MG-63 cells were treated with glycine for 10min and 0.5% Triton X-100 for 10min. The cells were then mixed with 4,6-diamidino-2-phenylindole (DAPI) in the dark for 30min, and images were captured by fluorescence microscopy.

### Morphological Assessment of the Different Bioactive Titanium Surfaces

Scanning electron microscopy was employed to observe the titanium disk morphologies. MG-63 cells were inoculated on both smooth and rough disks and cultured for 24h, followed by fixing with pre-cooled 3% glutaraldehyde overnight at 4°C. Next, each sample was fixed with pre-cooled 1% acetic acid at 4°C for 1h. The samples were then dehydrated for 10min using acetone/isoamyl acetate (1:1) and dehydrated for 30min with isoamyl acetate. Each dehydrated sample was immersed first in a 520% acetonitrile solution, followed by 15-min immersions in 70, 80, 90, 95, and 100% acetonitrile solutions, and a final transfer to a solution of 100% acetonitrile. The samples were then vacuum-dried for 30min and coated with carbon and gold particles for observation under SEM.

### Western Blot Analysis

MG-63 cells cultured on the different bioactive titanium disks under normoxia and hypoxia were collected and fully lysed using RIPA Lysis Buffer (Beyotime, China). Protein content was quantified using a bicinchoninic acid kit (Beyotime, China). After adjusting the sample concentrations, SDS-polyacrylamide gel electrophoresis (Amresco, OH, United States) was carried out using 30μg of protein per sample. The separated protein bands were then transferred to a nitrocellulose membrane and incubated in a blocking buffer solution containing 5% skimmed milk powder (Sangon Biotech, China) overnight at 4°C. Next, the membrane was washed with TBST, incubated with goat anti-human p-JAK1, p-STAT1, JAK1, STAT1, HIF-1α, BMP2, COL-1, and RUNX2 antibodies (Abcam, 1:1000, United States), and visualized using ECL reagent (Pierce, IL, United States). Membranes were cleared with a clearing buffer and re-incubated with GAPDH (Abcam, 1:5,000, United States) for visualization as an internal reference. The results were analyzed using the Chemi-Genius gel imaging system for the expression of the target protein.

### RT-qPCR

RNA was extracted using TRIzol and reverse-transcribed into cDNA using a reverse transcription kit (Tiangen, China) according to the manufacturer’s instructions. Next, the RNA content was detected using fluorescence (Bio-Rad, United States). The sequences of the RT-PCR primers used are listed in Table 1. The reaction cycle was carried out at 95°C for 20s, annealing at 60°C for 30s, and extension at 72°C for 30s. The results were calculated using the 2^-ΔΔCT^ method.

**Table 1:** Gene sequences.

Gene	Sequence (5'–3')
*BMP2*	ACCCGCTGTCTTCTAGCGTTTTCAGGCCGAACATGCTGAG
*COL1*	GAGGGCCAAGACGAAGACATCCAGATCACGTCATCGCACAAC
*RUNX2*	TGGTTACTGTCATGGCGGGTATCTCAGATCGTTGAACCTTGCTA
*GAPDH*	GGAGCGAGATCCCTCCAAAATGGCTGTTGTCATACTTCTCATGG

### Animal Experiments

The protocol for our animal experiments was approved by the Ethics Committee of Long Gui Xing Ke Animal Farm, Baiyun District, Guangzhou. Titanium implants from different groups (3.45mm×10mm) were implanted into four adult male New Zealand white rabbits (aged 20weeks and weighing approximately 3.0kg). Animal experiments were performed in a plain zone (Guangzhou, China) and a highland zone (Xizang, China). Before experiments, all animals were lived in the correspondent place at least 6months in the SPF environment. Animals were anesthetized using Sumian Xin (Animal Husbandry Research Institute, Jilin, China) at 0.15ml/kg body weight. Preoperatively, 0.6ml of Primacaine (Merignac Cedex, France; 0.2ml/kg of body weight) was locally injected into the surgical site of the tibia. The titanium plates were implanted approximately 7mm–12mm beneath the joint, and a similar operation was performed on the other side of the tibia. The rough plate was placed in one side, and the smooth plate was placed in other side. After the operation, all animals were injected with 0.3mg (Pharmaceutical Company, Sichuan, China) and allowed to move freely. At either 2 or 4weeks post-surgery (*n*=5 at each time point), the animals were sacrificed by excessive anesthetic (1.5ml/kg) and samples were collected for histological analysis. Bone grafts and soft tissues were collected and fixed in 10% neutral-buffered formalin (Cohen et al., 2020).

### Bone-to-Implant Contact Evaluation

Tissue specimens were dehydrated in a gradient dilution of ethanol, immersed in 100% resin, and embedded in methyl methacrylate. They were then cut in the buccolingual direction and parallel to the axis of the implants using a low-speed diamond saw (SP1600, Leica Biosystems, Germany). The section containing the implant was ground until its thickness reached 60–80μm. It was then stained with methylene blue-acid fuchsin. Each specimen was observed under an optical microscope (Olympus BX41, Olympus Co., Japan) and analyzed using the OsteoMeasure™ software. Bone-to-implant contact (BIC) was quantitatively measured as follows: BIC (%)=(sum of the length of BIC)/(circumference of the implant chamber region)×100. BIC was defined as the interface where the bone tissue was located within 20μm of the implant surface without any intervention of the soft tissue.

### Statistical Analysis

Each measurement was repeated in triplicate and averaged. Data were collected and reported as the mean±SD. One-way ANOVA was carried out between the compared groups, followed by the Student-Newman-Keuls method. All analyses were conducted using SPSS Statistics 17, and the significance level was set at 0.05.

## Results

### Morphological Characteristics of the Different Titanium Surfaces

**Figure 1:**
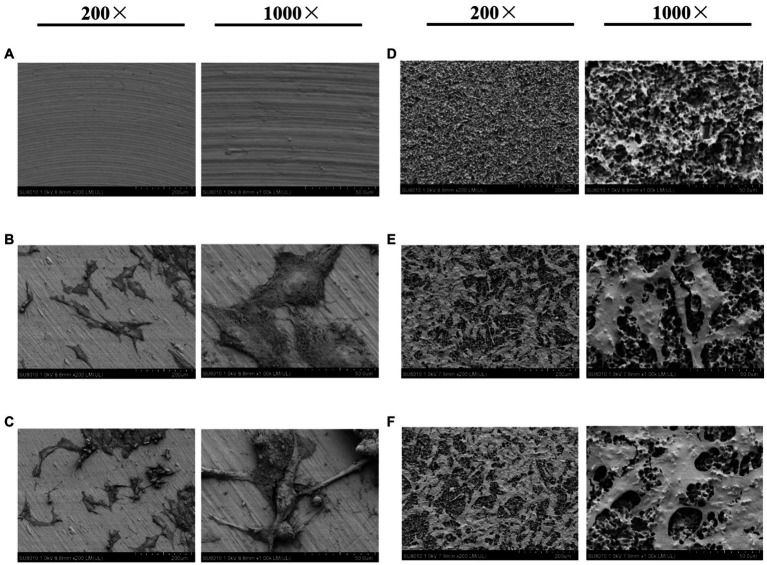
Scanning electron microscopy (SEM) images of titanium plates and osteoblasts. **(A)** Smooth titanium plate, **(B)** MG-63 cells on a smooth surface under normoxic conditions, **(C)** MG-63 cells on a smooth surface under hypoxic conditions, **(D)** Rough titanium plate, **(E)** MG-63 cells on a rough surface under normoxic conditions, and **(F)** MG-63 cells on a rough surface under hypoxic conditions.

Scanning electron microscopy analysis showed that mechanical polishing resulted in a smooth, “mirror-like” titanium surface (Figure 1A), whereas the sandblasted and acid-etched surface was rough and displayed a “honeycomb” structure with different porosities (Figure 1D). MG-63 cells were inoculated on the two titanium surfaces. Under normoxic or hypoxic conditions, titanium disks with a smooth surface contained numerous cells connected by cell matrix that grew in a single layer and with a flat appearance (Figures 1B,C). Conversely, under normoxic and hypoxic conditions, disks with a rough surface were covered with multiple cell layers connected by the matrix, with fractures at multiple sites, unclear boundaries, and unevenness (Figures 1D–F).

### Titanium Plates Promote Cell Proliferation Under Hypoxia

**Figure 2:**
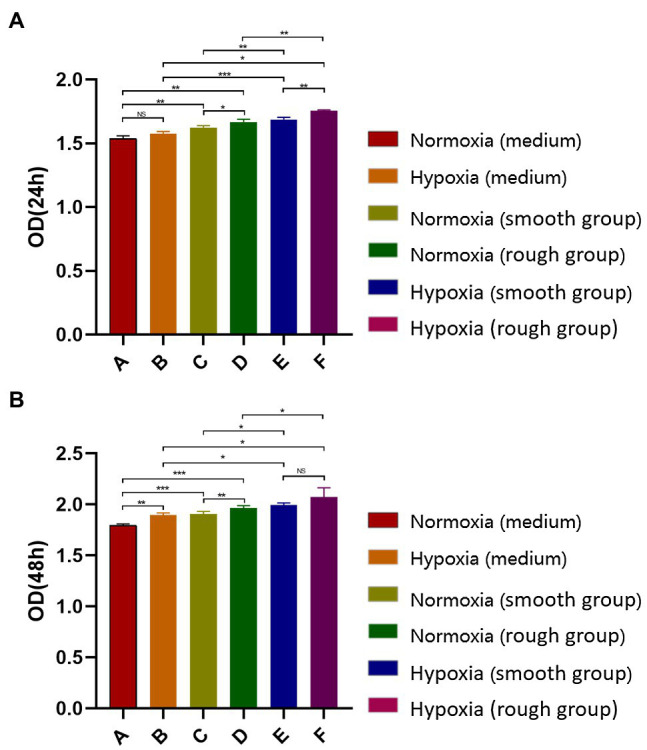
Detection of MG-63 cell proliferation rate using a CCK-8 kit. **(A)** MG-63 cell proliferation rate at 24h and **(B)** MG-63 cell proliferation rate at 48h. NS, no significance; ^*^p<0.05, ^**^p<0.01, and ^***^p<0.001.

**Figure 3:**
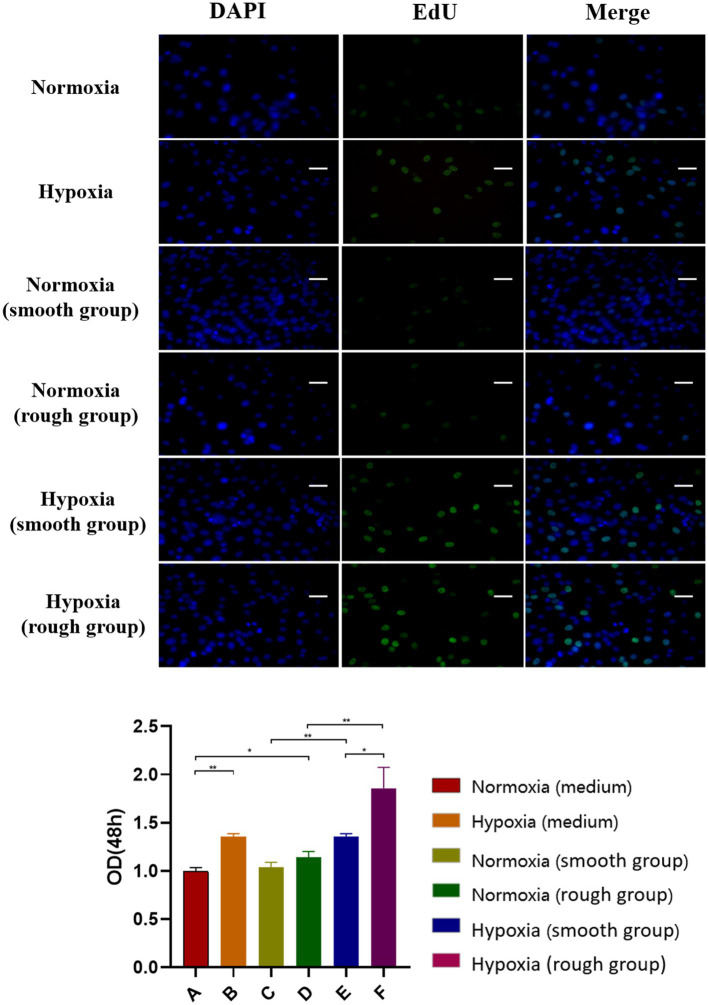
EdU staining results for the different groups. The EdU staining results and quantification of different groups under different environment [Blue: 4,6-diamidino-2-phenylindole (DAPI), Green: EdU]. Scale bar=200μm. ^*^p<0.05, ^**^p<0.01.

At both 24 and 48h, the hypoxic condition was better than the normoxic one in term of proliferation (*p*<0.05; Figures 2A,B). After 48h, the EdU assay showed that cell proliferation was significantly higher under hypoxia than normoxia. Moreover, cell viability was higher on titanium disks with a rough surface than on those with a smooth surface (Figure 3). Our findings suggest that hypoxic conditions might benefit cell proliferation. Additionally, the two surface treatments improved cell proliferation, with a particularly significant affect induced by the rough surface.

### A Rough Surface Enhances Osteogenic Differentiation Under Hypoxia

**Figure 4:**
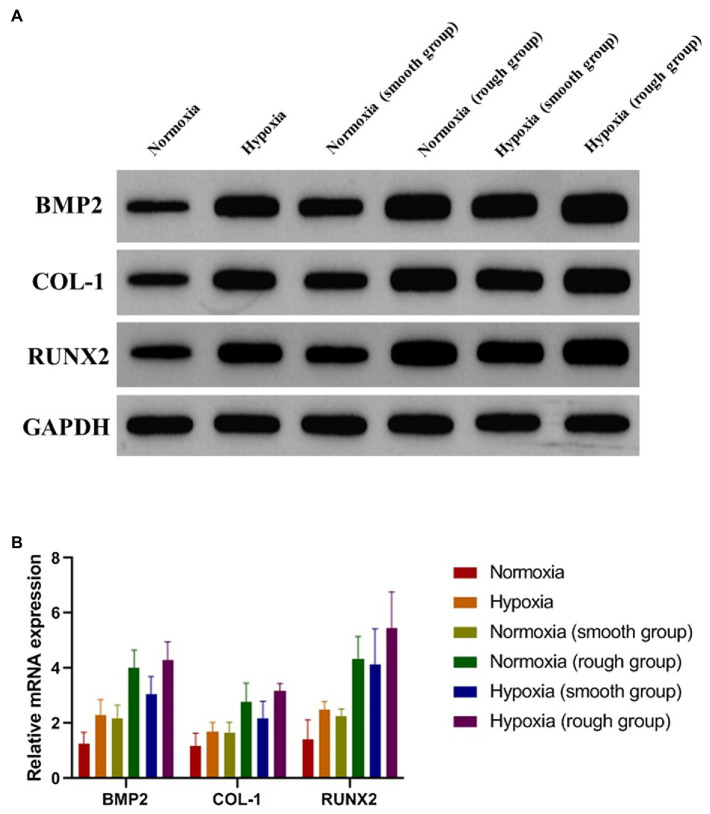
Osteogenic-related proteins and mRNA (BMP2, COL-1, and RUNX2) expression of MG-63 cells in different groups. **(A)** Western blotting and **(B)** RT-qPCR. ^*^p<0.05, ^**^p<0.01, and ^***^p<0.001, ^*^compared with the corresponding group maintained under normoxic conditions.

To evaluate the osteogenic differentiation of MG-63 cells in environments with different oxygen levels and on different surfaces, the expression of osteogenic-related proteins was detected 14days after culture. The results showed that the expression of osteogenic-related proteins was promoted under hypoxic conditions and further enhanced by the rough surface (Figure 4A; BMP2, *p* <0.05; COL-1 *p* <0.05; RUNX2, *p* <0.01). These results were confirmed by the RT-qPCR findings with the rough surface found to increase the expression of genes associated with osteogenesis in MG-63 cells (Figure 4B).

### JAK1/STAT1/HIF-1α Pathway Activation Under Different Conditions

**Figure 5:**
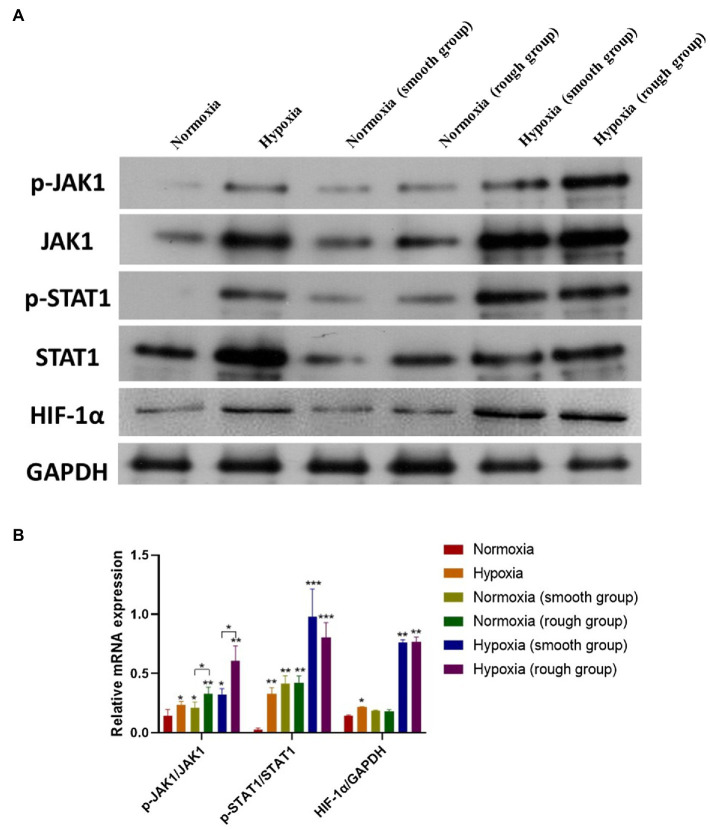
The JAK1/STAT1/HIF-1α pathway activation in different groups. **(A)** Protein expression of p-JAK1, JAK1, p-STAT1, STAT1, HIF-1α, and GAPDH; **(B)** Relative protein expression of p-JAK1/JAK1, p-STAT1/STAT1, and HIF-1α/GAPDH. ^*^p<0.05, ^**^p<0.01, and ^***^p<0.001, ^*^compared with the corresponding group maintained under normoxic conditions.

From the western blot analysis of MG-63 cells, we determined that the JAK1/STAT1/HIF-1α pathway displayed a reduced expression (p-JAK1/JAK1, p-STAT1/STAT1, and HIF-1α/GADPH) under normoxic conditions, but was activated under hypoxic conditions (Figures 5A,B). Although the rough surface promoted activation of the JAK1/STAT1/HIF-1α pathway (p-JAK1/JAK1, p-STAT1/STAT1, and HIF-1α/GADPH) under normoxic conditions, no significant differences were observed in the activation of this pathway between the smooth surface and rough surface groups under hypoxia (Figures 5A,B).

### Histological Analysis

**Figure 6:**
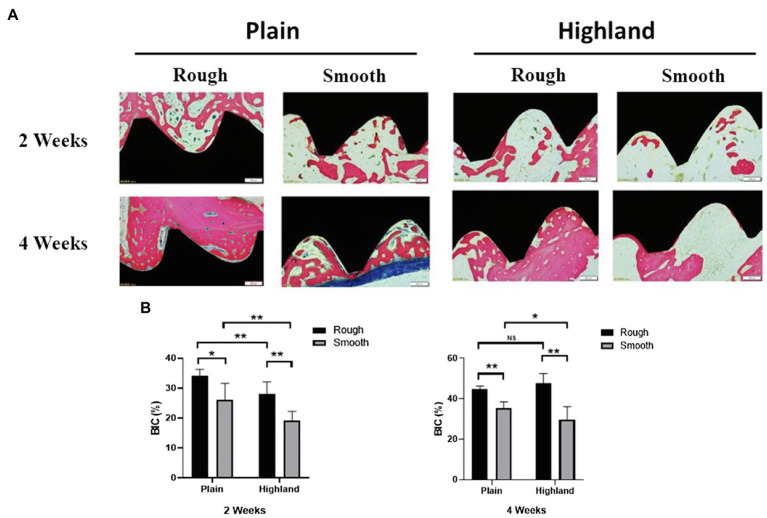
Histological observation of implants and the surrounding tissues [methylene blue-acid fuchsin **(A)**]; comparison of Bone-to-Implant Contact (BIC; %) at week 2 and 4 **(B)**. NS, no significance; ^*^p<0.05, ^**^p<0.01.

Data collected from our animal-based experiments showed that no implant loss occurred at 2 and 4weeks post-operation. In the high-altitude hypoxic environment, new bone formation was detected on all of the implants, and osseointegration was observed. Compared to implants with a smooth surface, those with a rough surface showed more bone regeneration at 2weeks post-operation. Intimate bone contact with the implant surface was observed in the healing cavity around the implants; only a small amount of new bone tissue was found on implants with a smooth surface. At 4weeks in both high-altitude and plain area, new bone covered most of the implants with rough surfaces, whereas relatively few covered areas were detected on implants with smooth surfaces (Figure 6A). By comparing the BIC (%) of each group, we noted that at week 2, implants with a rough surface showed superior bone formation and osseointegration capacity compared to those with a smooth surface, under both normoxic and hypoxic conditions (Figure 6B). At week 4, significant differences were detected between smooth and rough surface both in plain and highland. Titanium plates with a rough surface had significantly better osteogenesis to the hypoxic microenvironment compared to those with a smooth surface and were able to regenerate and bind bone in a hypoxic environment.

## Discussion

Titanium and its alloys are metal materials commonly used in the field of biomedicine (He et al., 2020; Tan et al., 2020), more specifically in the treatment of bone defects, as implant devices to replace damaged or defective tissues. Recent research on titanium surface modifications has focused primarily on slurry spray coating, hydroxyapatite coating, micro-arc oxidation, laser irradiation, and anodic oxidation (Liu et al., 2017; He et al., 2019; Pippenger et al., 2019). Moreover, studies have reported that surface treatment of uncoated grafts is superior to that of coated grafts, for example, in terms of outstanding biocompatibility and osteoconductive properties (Horvathy et al., 2016; Wu et al., 2018a). However, bone formation is not only affected by bone materials, but also by O2 concentration. Indeed, hypoxic environments can promote bone formation and bone healing (Xue et al., 2020; Yu et al., 2020). Therefore, in the present study, we explored the activities of osteoblasts on the surface of different bioactive titanium implants under normoxic and hypoxic conditions.

Currently, sandblasting and acid etching (SLA) is one of the most widely used non-coating surface treatment methods (Corvino et al., 2020; Lu et al., 2020). SLA combines the advantages of physical and chemical etching, while also offering an inexpensive and simple platform (Lu et al., 2020). In our study, irregular honeycomb-shaped holes were formed on the surface of the titanium plate following SLA. Considering that cell adhesion is a prerequisite for the proliferation and differentiation of bone cells, the surface morphology of biomaterials directly affects early adhesion (Kim et al., 2019). Studies have confirmed that SLA not only effectively increases the contact area of osteoblasts, but also enhances their adhesive and proliferative capacity (Bayram et al., 2012; Ding et al., 2015). The results of the CCK-8 and EdU analyses in the current study support that rough surface promotes cell proliferation under hypoxia. Moreover, at 2weeks, the expression of osteogenic-related proteins and genes (BMP2, COL-1, and RUNX2) was promoted under hypoxic conditions (Figure 4). The rough surface further enhanced osteogenic differentiation and hypoxia enhanced the expression of JAK1 and STAT1.

The JAK1/STAT1 pathway plays an integral role in the transmission of hypoxia signals and mediates the effects of hypoxia (Yang et al., 2017; Wu et al., 2018b; Liu et al., 2020b). Moreover, mesenchymal stem cells ameliorate hypoxia and reoxygenation injury in renal tubular epithelial cells through the JAK/STAT signaling pathway (Zhang et al., 2017). In addition, activation of the JAK1/STAT1 pathway can promote HIF-1α expression in hypoxic environments (Yang et al., 2017). Similarly, in our study, activation of the JAK1/STAT1 pathway stimulated HIF-1α expression by western blotting. Overexpression of HIF-1α can upregulate the vascular endothelial growth factor, induce neovascularization, and accelerate bone formation (Du et al., 2018; Liu et al., 2019). Therefore, hypoxia can enhance osteogenic differentiation *via* the JAK1/STAT1/HIF-1α pathway.

Our animal model revealed that bone formation under high-altitude hypoxic conditions was significantly slower than that under normoxia; this result was consistent with previous studies (Lezon et al., 2016; Suresh et al., 2019). In highland environments, the extent of osteogenesis on both rough and smooth surfaces was lower than that observed in plains at 2weeks. However, no significant difference was observed in the BIC for the rough surface between the two conditions at 4weeks. Moreover, the roughness of the implant surface was positively associated with the amount of newly generated bone. Studies have reported that high-altitude hypoxic environments delay the healing of bone tissue and produce a relatively insufficient mass of new bone, while also affecting the osseointegration efficiency of the implant (Shan et al., 2017; Uhl, 2018). In contrast, hypoxic conditions promote osteogenic differentiation *in vitro*. This difference may be the result of a complex *in vivo* environment. For example, in addition to hypoxic conditions, low-pressure conditions also play an important role in osteogenesis *in vivo* (Wang et al., 2006; Torigoe et al., 2007). In fact, a low-pressure system has been shown to facilitate the perfusion of a larger number of mesenchymal stem cells into the porous scaffold, enhancing bone formation within the composites (Torigoe et al., 2007). The difference of *in vitro* and *in vivo* condition caused the discrepancy of osteogenesis results under normoxia and hypoxia environment. To further assess these effects, our future work will include constructing a low-pressure model and determining how different titanium surfaces affect osseointegration.

Collectively, the findings of this study indicate that rough surfaces more effectively promote bone formation in the plains and highlands compared to smooth surfaces. Moreover, under hypoxic conditions, implants with a rough surface enhanced the efficiency of bone binding at an early stage, that is, both at 2 and 4weeks. However, no significant difference was detected between the two conditions in implants with a rough surface at 4weeks, suggesting that implant loading should be performed after 4weeks in highland environments.

## Conclusion

Compared to a smooth surface, a rough surface was more conducive to the proliferation of osteogenesis and implant osseointegration. The early enhanced osteogenic differentiation detected on tibial implants under hypoxic conditions appears to be associated with upregulation of the JAK1/STAT1/HIF-1α pathway. Meanwhile, long-term observation showed that hypoxia inhibited bone formation and osseointegration.

## Data Availability Statement

The raw data supporting the conclusions of this article will be made available by the authors, without undue reservation.

## Ethics Statement

The animal study was reviewed and approved by Ethics Committee of Long Guixingke Animal Farm, Baiyun District, Guangzhou.

## Author Contributions

MR, YW, and PC conceived and designed the study. YW, ZG, HL, ZL, PS, JZ, WY, HC, RY, and YY performed the experiments and analyzed the data. YW wrote the manuscript. YW, ZG, PC, and MR revised the manuscript. All authors contributed to the article and approved the submitted version.

## Funding

This study was funded by the Natural Science Foundation of Guangdong Province (grant number 2016A030310240), the Guangdong Science and Technology Innovation Strategy Special Fund Project (grant number 2018KJY2014), the Medical Research Foundation of Guangdong Province (grant number B2019117), the Guangdong Provincial Administration of Traditional Chinese Medicine, Chinese Medicine Research Project (grant number 20202129), the Natural Science Foundation of Tibet Autonomous Region (grant number XZ2017ZR-ZYZ37), and the National Natural Science Foundation of China for Youth (grant number 81600900).

## Conflict of Interest

The authors declare that the research was conducted in the absence of any commercial or financial relationships that could be construed as a potential conflict of interest.

## Publisher’s Note

All claims expressed in this article are solely those of the authors and do not necessarily represent those of their affiliated organizations, or those of the publisher, the editors and the reviewers. Any product that may be evaluated in this article, or claim that may be made by its manufacturer, is not guaranteed or endorsed by the publisher.
